# Delta and theta eeg activity during resting state is altered in patients affected by major depression

**DOI:** 10.1192/j.eurpsy.2021.908

**Published:** 2021-08-13

**Authors:** C. Spironelli, F. Fusina, A. Angrilli

**Affiliations:** 1 Padova Neuroscience Center, Università degli Studi di Padova, Padova, Italy; 2 Department Of General Psychology, Università degli Studi di Padova, Padova, Italy

**Keywords:** Major Depression, EEG bands, resting state, psychophysiology

## Abstract

**Introduction:**

Major depression (MD) is associated with cognitive and behavioral alterations in many domains. It is not well clear what cortical structures and functional alterations characterize MD patients during resting state, a condition during which mind wandering process is prevailing.

**Objectives:**

In MD patients with severe levels of depression we expected, during resting state, an altered asymmetry of cortical activity in the EEG bands that generally mark neurological impairment, i.e. Delta and Theta EEG bands.

**Methods:**

30 MD patients under pharmacological treatment and 32 matched controls underwent an EEG recording (38 scalp sites) during 5 min resting state with open eyes. Eye movements were corrected by ICA modeling and the 5 min recording was divided in 2 sec epochs from which Delta and Theta spectral powers were extracted.

**Results:**

Spectral analysis of the 5 min resting state revealed a significant difference between the two groups at the level of left temporal lobe. MD patients showed larger Delta and Theta spectral power in the left superior temporal gyrus at the level of Brodmann’s Areas 22 and 42.

**Conclusions:**

Results evidenced a cortical inhibition (greater EEG Delta and Theta activity) in left temporal linguistic areas in severe depression, a result pointing to a different mind wandering process and thought architecture in MD patients during resting state.

